# Supplementary material for: Resilience to periodic disturbances and the long-term genetic stability in Acropora coral
Source: Commun Biol. 2024 Apr 4;7:410. doi: 10.1038/s42003-024-06100-0 (PMC10995172; doi:10.1038/s42003-024-06100-0)
Supplement: Supplementary file 2 — Supplementary Information [file 42003_2024_6100_MOESM2_ESM.pdf]

Supplementary Information

Resilience to periodic disturbances and the long-term genetic stability in Acropora coral

Thomas L[1,2], Şahin D[1,2], Adam AS[1], Grimaldi CM[1,2], Ryan N[1], Duffy S[1,2], Underwood JN[1] , Kennington WJ[2,3], Gilmour JP[1,2]

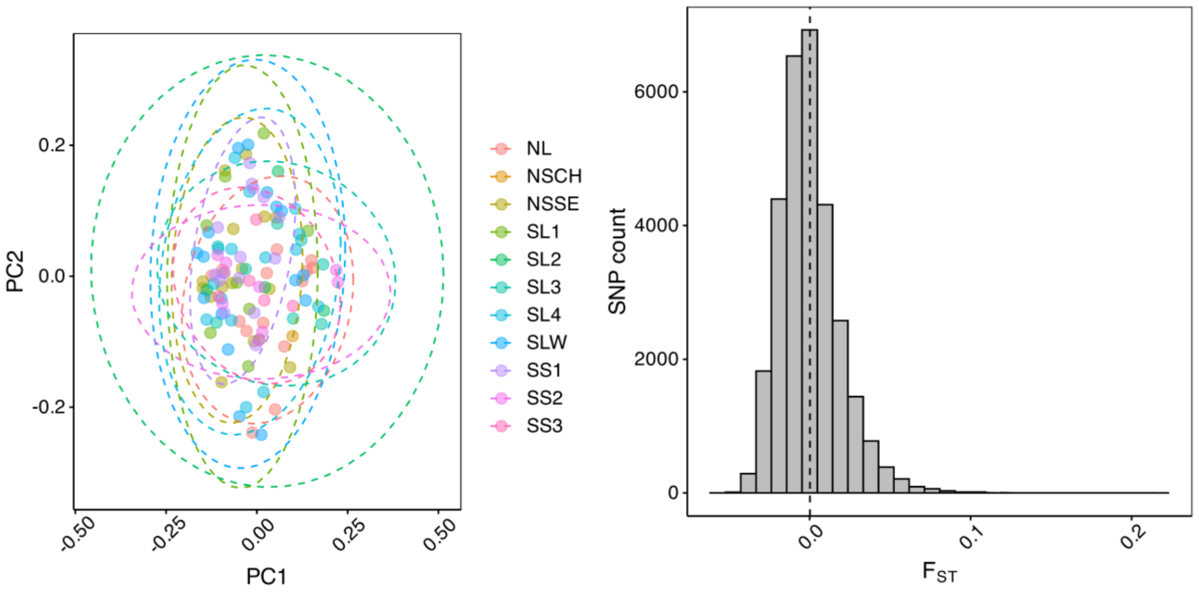

Figure S1 Left: Scatter plot of the first two principal components generated using PCAangsd based on a reduced dataset of 29,975 SNPs (mean 10x coverage). Each point represents a different coral colony colour coded by site of collection. Right: Histogram of genome-wide estimates of F_{ST} based on the reduced SNP dataset for the 2021 WGS dataset. Vertical line denotes mean value of -0.0002.

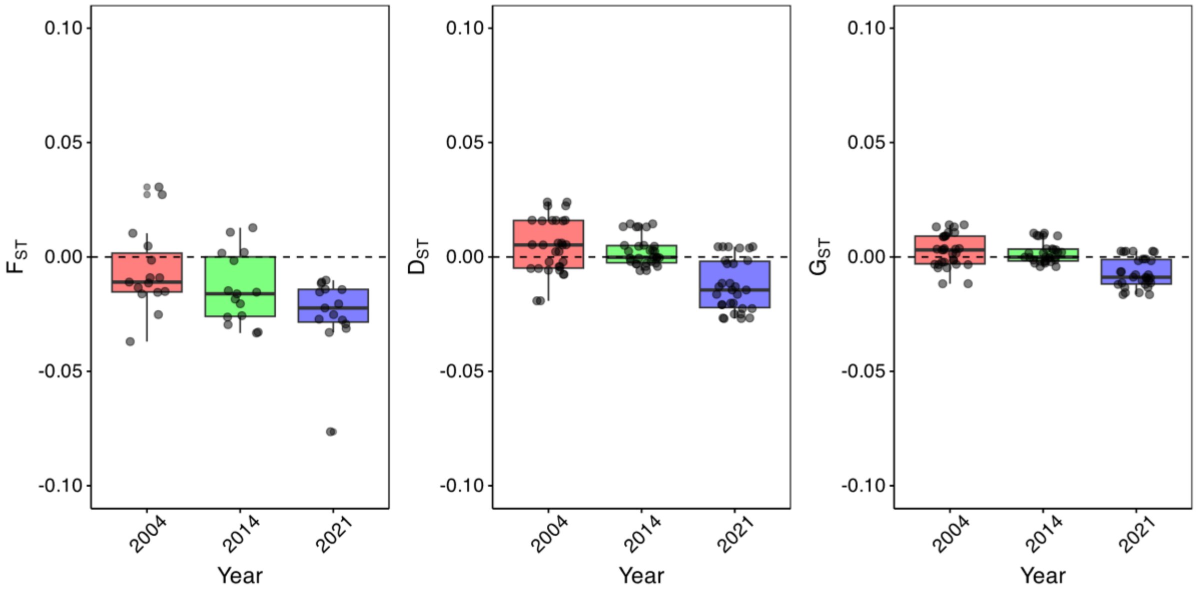

Figure S2 Estimates of genetic differentiation among years for three common metrics (F_{ST}, G_{ST}, D_{ST}) for the same 6 LTM sites sampled in 2004, 2014, and 2021. Time points for which only a subset of sites were sampled were removed (e.g. 2009 and 2015).

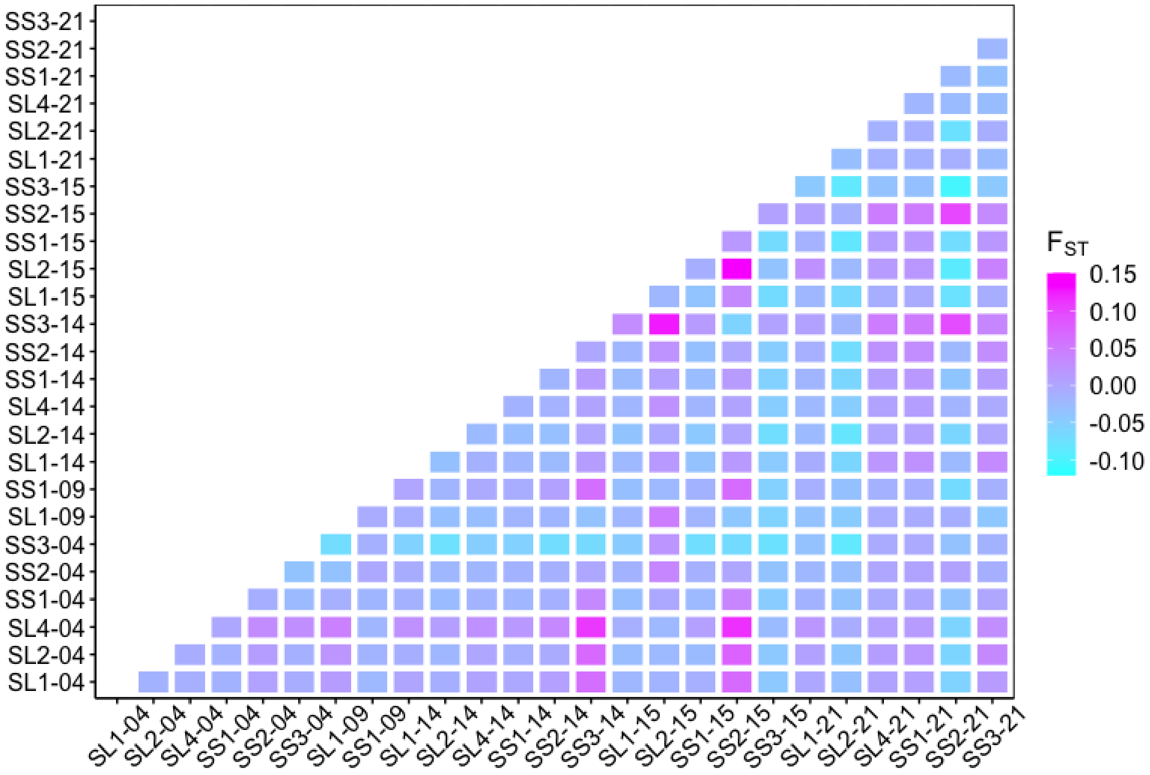

Figure S3 Top: Heatmap of pairwise F_{ST} across all site/timepoint comparisons. Warmer colours denote high values. See Table S2 for values.

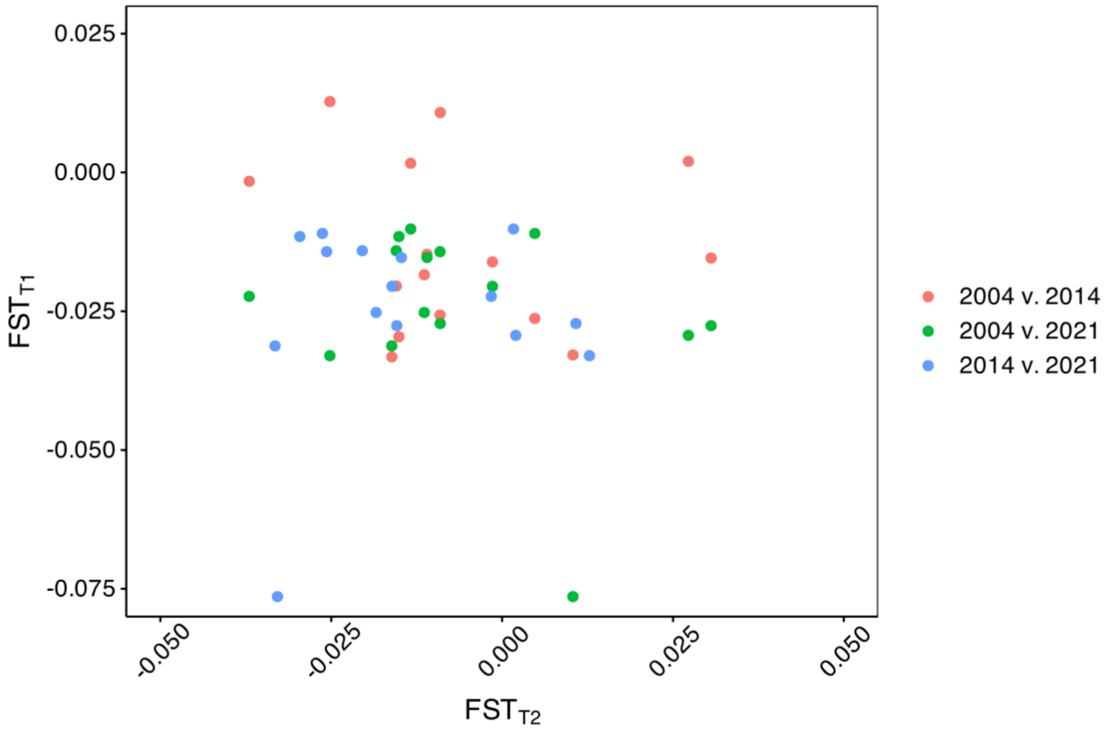

Figure S4 Regression of genetic differentiation (F_{ST}) for different site:timepoint comparisons (P=0.58). Each point represents a pairwise comparison at different time points.

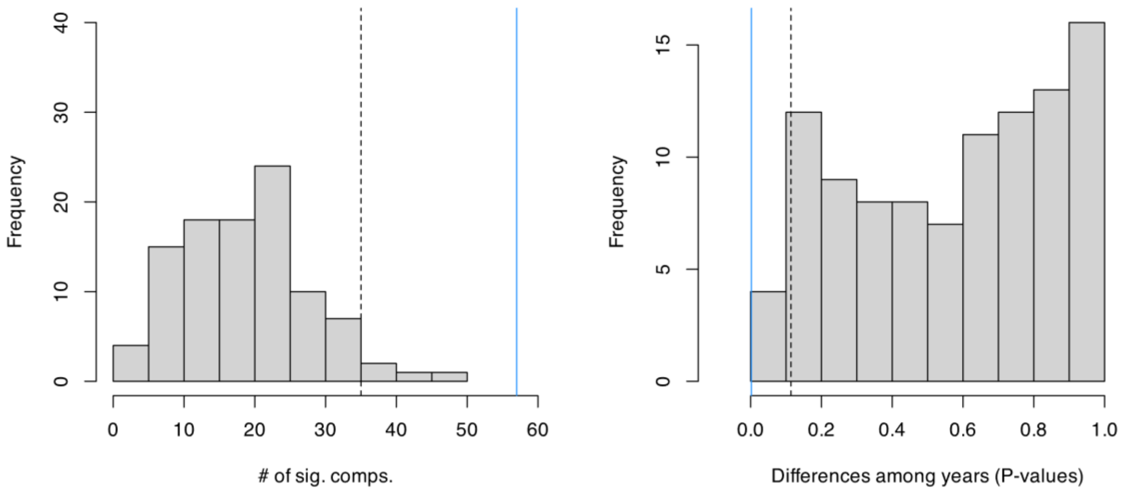

Figure S5 Results from randomization trials where site:year assignments were shuffled and genetic differentiation among sites and years recalculated. Left: histogram of significant pairwise FST comparisons among sites across 100 bootstrap replicates. Right: histogram of p-values for estimates of genetic variation among timepoints under the AMOVA framework after 100 bootstrap replicates. Vertical black lines denote 95% tails of the randomized distribution and blue lines represent the observed values.

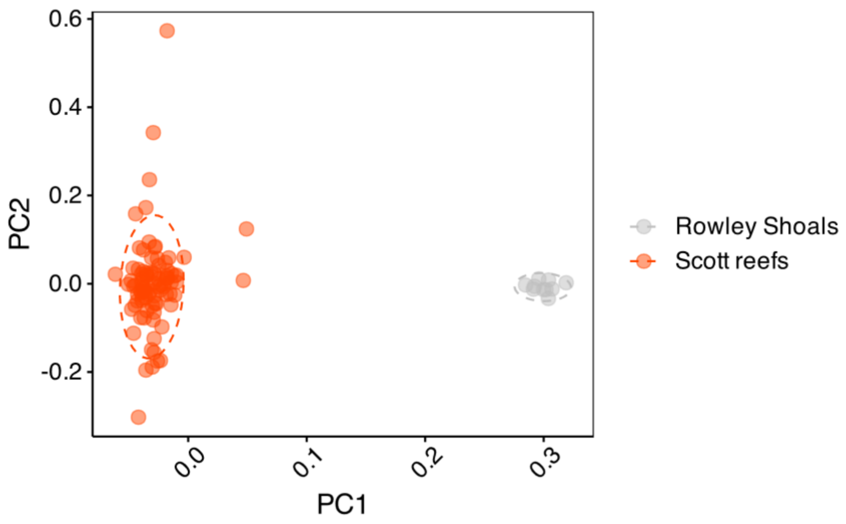

Figure S6 Scatter plot of the first two principal components based on whole genome resequencing across 7,476,225 variant sites using samples from Scott reef (n=111, blue) and Rowley Shoals (n=10, grey).

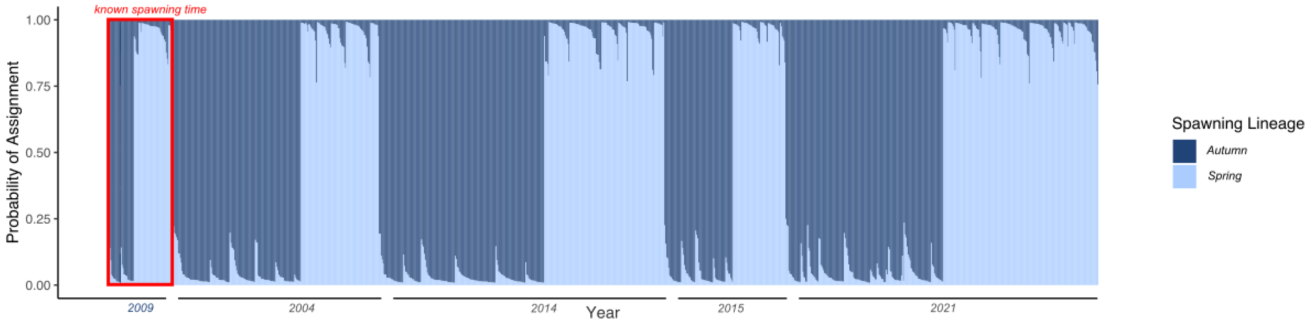

Figure S7 Bayesian admixture plot for all samples collected over the 5 timepoints. Samples are clearly separated into two diverged spawning lineages. Samples from 2009 had known seasonal spawning phenotypes based on multiyear surveys that were used to assign samples from other years to a spawning lineage.

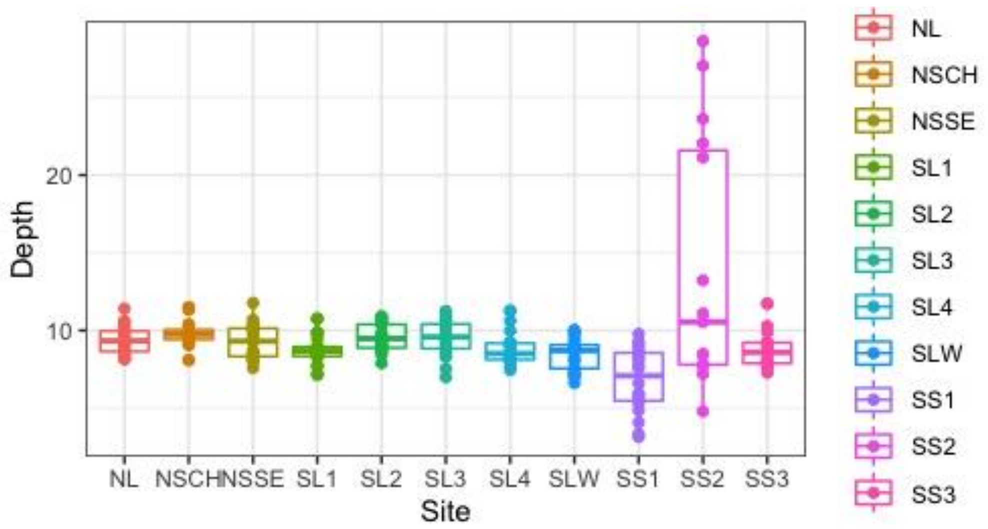

Figure S8 Boxplot of genome-wide coverage for samples from Scott reef (2015, n=8) and Rowley Shoals (2017, n=9) used to estimate divergence among reefs and shifts in genetic diversity based on genotype likelihoods.

Supplementary Note 1 (Mapping with bwa-mem)

```
#!/bin/bash
CHUNK=$2
COUNTER=0
FQ="${@:3}"

for i in $FQ; do
   if [ $COUNTER -eq 0 ]; then
   echo -e "#!/bin/bash\n#SBATCH --ntasks=1\n#SBATCH --cpus-per-task=12\n#SBATCH \
    -t 24:00:00\n#SBATCH --mem 24000" > TEMPBATCH.sbatch; fi
    BASE=$( basename $i _R1.fastq.gz )
   echo "module load bwa" >> TEMPBATCH.sbatch
   echo "bwa mem $1 ${BASE}_R1.fastq.gz ${BASE}_R2.fastq.gz > ${BASE}.sam" >> TEMPBATCH.sbatc
h
   echo "module load samtools" >> TEMPBATCH.sbatch
   echo "samtools view -bSq 10 ${BASE}.sam > ${BASE}_BTVS-UNSORTED.bam" >> TEMPBATCH.sbatc
h
   echo "samtools sort ${BASE}_BTVS-UNSORTED.bam > ${BASE}_UNDEDUP.bam" >> TEMPBATCH.sb
atch
   echo "module load java" >> TEMPBATCH.sbatch
   echo "java -Xmx4g -jar picard.jar MarkDuplicates REMOVE_DUPLICATES=true \
   INPUT=${BASE}_UNDEDUP.bam OUTPUT=${BASE}.bam METRICS_FILE=${BASE}-metrics.txt \
   VALIDATION_STRINGENCY=LENIENT" >> TEMPBATCH.sbatch
   echo "samtools index ${BASE}.bam" >> TEMPBATCH.sbatch
   echo "rm ${BASE}.sam" >> TEMPBATCH.sbatch
   echo "rm ${BASE}_BTVS-UNSORTED.bam" >> TEMPBATCH.sbatch
   echo "rm ${BASE}_UNDEDUP.bam" >> TEMPBATCH.sbatch
   let COUNTER=COUNTER+1
   if [ $COUNTER -eq $CHUNK ]; then
   sbatch TEMPBATCH.sbatch
   COUNTER=0; fi
```

```
done
if [ $COUNTER -ne 0 ]; then
sbatch TEMPBATCH.sbatch; fi
```

Supplementary Note 2 (Sequencing coverage)

```
#!/bin/bash
samtools depth -a -f $1.bamlist > $1.coverage.txt
awk '{sum=0; for (i=3; i<=NF; i++) { sum+= $i } print sum}' all.coverage.txt | \
awk '{ sum += $1 } END { if (NR > 0) print sum / NR }' > $1.mean.txt
```

Supplementary Note 3 (Call single nucleotide polymorphisms)

```
!/bin/bash

bcftools mpileup -f aten.chr.fasta -d 50 -C50 --per-sample-mF --annotate FORMAT/AD,FORMAT/ADF,FORMAT/ADR,FORMAT/DP,FORMAT/SP,INFO/AD,INFO/ADF,INFO/ADR --redo-BAQ --min-BQ 20 -b autumn.bamlist -q 20 -Q 20 -Ou --threads 30 | bcftools call --skip-variants indels -mv -Ov -o autumn_snps.vcf --threads 30
vcftools/src/cpp/vcftools --vcf autumn_snps.vcf --max-missing .90 --maf 0.05 --recode --recode-INFO-all --out autumn_snps
bcftools filter -e 'MEAN(FMT/DP) < 10' autumn_snps.recode.vcf -o autumn_snps.filtered.vcf
vcftools --vcf autumn_snps.filtered.vcf --012 --out autumn_snps.filt
```

Supplementary Note 4 (Allele frequency spectrum and genetic diversity)

```
#!/bin/bash

/angsd -bam {pop.bamlist} -ref {reference}.fasta -anc {reference} -out {pop}.unfiltered \
   -uniqueOnly 1 -remove_bads 1 -only_proper_pairs 1 -trim 0 -C 50 -baq 1 \
   -minMapQ {INT} -minQ {INT} -minInd {INT} -setMinDepth {INT} -setMaxDepth {INT} \
   -GL 1 -doSaf 1 -doCounts 1 -P 16

/realSFS {pop}.unfiltered.saf.idx -fold 1 > {pop}.unfiltered.sfs

/realSFS saf2theta {pop}.unfiltered.saf.idx -sfs {pop}.unfiltered.sfs \
  -outname {pop}.unfiltered.out

/thetaStat do_stat {pop}.unfiltered.out.thetas.idx \
  -win 1000 -step 1000 -type 0 -outnames {pop}.slidingwindow.theta
```
