# Supplementary material for: Resilience to periodic disturbances and the long-term genetic stability in Acropora coral
Source: Commun Biol. 2024 Apr 4;7:410. doi: 10.1038/s42003-024-06100-0 (PMC10995172; doi:10.1038/s42003-024-06100-0)
Supplement: Supplementary file 3 — Description of Additional Supplementary Files [file 42003_2024_6100_MOESM3_ESM.pdf]

Description of Additional Supplementary Files

File name: Supplementary Data 1
Description: Pairwise FST (Weir and Cockerham 1984) values for the 2021 WGS dataset and based on 29,462 diploid SNPs with a minimum depth of 10 and minor allele frequency greater than 0.05. Significance levels are provided above the diagonal.

File name: Supplementary Data 2
Description: Pairwise FST (Weir and Cockerham 1984) values for the temporal microsatellite dataset. Significance levels are provided above the diagonal.

File name: Supplementary Data 3
Description: **R**esults from analysis of molecular variance (AMOVA) using the temporal microsatellite dataset and testing for hierarchical patterns of genetic structure among years, sample sites, and samples.

File name: Supplementary Data 4
Description: Sample collection and descriptive statistics for microsatellite data (averaged across loci) for each sample site at each time point: Inbreeding coefficient (FIS), observed (HO) and expected heterozygosity (HE), allelic richness (Ar), sample size (N) and unique multi-locus genotypes (MLGs).

File name: Supplementary Data 5
Description: Sample collection details for each timepoint and site. In total 986 colonies of Acropora sp. (previously Acropora tenuis) were collected from five timepoints (2004, 2009, 2014, 2015, and 2021) and from up to 6 long-term monitoring sites (SL1, SL2, SL4, SS1, SS2, SS3).
